# Mediating Role of Moral Disengagement Mechanisms in the Relationship Between Perceived Parental Warmth and Youth Violence

**DOI:** 10.3390/children12020246

**Published:** 2025-02-18

**Authors:** María J. Navas-Martínez, Lourdes Contreras, M. Carmen Cano-Lozano

**Affiliations:** Department of Psychology, University of Jaén, 23071 Jaén, Spain; lmcontre@ujaen.es (L.C.); mccano@ujaen.es (M.C.C.-L.)

**Keywords:** child-to-parent violence, peer violence, dating violence, moral disengagement, parental warmth

## Abstract

**Background/Objectives:** Although child-to-parent violence (CPV), peer violence (PV), and dating violence (DV) share risk factors and tend to co-occur, little is known about the common and differential mechanisms involved in the development of these types of youth violence. This study aims to (1) analyze the relationship between youth violence (CPV, PV, and DV) and perceived parental warmth and moral disengagement mechanisms and (2) explore the mediating role of moral disengagement mechanisms in the relationship between the lack of perceived parental warmth and youth violence. **Methods**: A cross-sectional population-based survey study was conducted. The sample consisted of 2124 Spanish adolescents (57.9% girls) aged between 13 and 17 years from educational centers. **Results**: The lack of perceived parental warmth (parental criticism-rejection in particular) is a common risk factor for all three types of violence and, more relevantly, is related to youth violence through moral disengagement mechanisms, highlighting a differential contribution of these mechanisms according to the type of violence. In particular, advantageous comparison and attribution of blame are specific mediators of CPV, and moral justification and distortion of consequences are specific to PV, whereas euphemistic language seems to be a common mediator of PV and DV. **Conclusions**: This study suggests that adolescents who perceive a lack of parental warmth are more likely to develop different dysfunctional cognitive mechanisms, which in turn are related to different types of youth violence. It would be important to promote warm parenting practices and address the cognitive mechanisms underlying youth violence.

## 1. Introduction

There is great social concern about youth violence, which is becoming more severe and frequent in more social contexts. In parallel, there is abundant scientific literature on the risk and protective factors involved in the development of violent behavior in adolescents (e.g., [1,2,3]).

Youth violence can manifest in different forms and across different interpersonal contexts. One type of youth violence that occurs within family relationships is violence by son and daughter toward parents, known as child-to-parent violence (hereafter CPV), which involves “any act of a child that is intended to cause physical, psychological or financial damage to gain power and control over a parent” [4] and dominate them [5] in a conscious, intentional and repeated manner over time [6]. In the context of peer relationships, peer violence (hereafter PV) is described as a pattern of repeated and deliberate aggression characterized by an imbalance of power between the one who perpetrates the violence and the one who suffers it [7], and where aggressors exhibit dominance-submission behavior patterns toward their victims [8]. Another interpersonal context in which youth violence occurs is in dating relationships. Dating violence (hereafter DV) is understood as physical, sexual, or psychological abuse of a partner, including threats [9].

It has been observed not only that the rates of these types of youth violence are high but also that they frequently co-occur [10,11,12] and are interrelated [13]. Likewise, multiple risk factors for CPV, PV, and DV have been identified, many of which are common among these three types of violence, such as dysfunctional family dynamics [14,15,16]. However, few studies have explored the mechanisms involved in the relationship between the risk factors of youth violence and its development, and even fewer have explored if these mechanisms differ depending on the type of violence. For this reason, the purpose of this study was to analyze the influence of the lack of perceived parental warmth on youth violence (CPV, PV, and DV) through the cognitive mechanisms of moral disengagement and to compare whether these variables play a differential role depending on the type of youth violence.

### 1.1. Parental Warmth and Youth Violence

Research has consistently highlighted that parental warmth plays a crucial role in children’s psychological adjustment across cultures, while its absence has been linked to both internalizing and externalizing behavioral problems [17,18,19]. A frequently identified family characteristic associated with different types of youth violence is the lack of parental warmth [14,20]. According to the evidence-based Parental Acceptance-Rejection Theory (IPARTheory) [17], parental relationships can be assessed along an acceptance-rejection continuum on which individuals are placed according to their perceived relationship history with their caregivers. Variables such as parental warmth-communication represent the positive end of the theoretical continuum of parental acceptance-rejection and include behaviors such as displays of affection, expressions of emotional support, and effective and respectful communication with children [21,22]. At the other extreme, variables such as parental criticism-rejection represent the negative end of the continuum and refer to behaviors that include constant disapproval, expressions of hostility, and the perception of being a burden to parents [23].

In the field of CPV, parental warmth during childhood acts as a protective factor against physical CPV [24]. Among judicial samples, one of the variables that differentiates CPV offenders from other offenders is the lack of parental warmth. Specifically, the offenders with CPV crimes perceived low levels of parental warmth-communication and higher levels of parental criticism-rejection compared to the offenders with other crimes [25,26]. Maternal emotional rejection is related to CPV [20] and increased rebellious behaviors towards mothers [27]. More importantly, low levels of warmth-communication and high levels of criticism-rejection significantly increase CPV behaviors [28], leading to the suggestion that family affectivity is a key aspect of violence prevention [29].

Regarding PV, consistent with the above, Buelga et al. [30] found that a family climate characterized by open and empathic communication can prevent PV, indicating that parental warmth would also play a protective role in this type of violence [31]. It has been found that parental rejection is associated with PV behaviors, confirming that adolescents who feel rejected by their families tend to engage in more PV-related behaviors, whereas parental warmth is negatively associated with PV [32]. Furthermore, adolescents involved in PV report low levels of maternal warmth and high levels of rejection, particularly from fathers [33]. In line with this, Nocentini et al. [16] concluded in their systematic review that parental warmth and affection were negatively associated with PV in 85% of the studies analyzed. In fact, low parental acceptance and involvement have been identified as significant risk factors for PV [34], although more studies are needed to examine the predictive power of the lack of parental warmth in this type of violence.

The scientific literature concerning DV also underscores the importance of family-of-origin factors (e.g., attachment, witnessing violence between parents, poor parenting, parental support) in this type of violence (e.g., review by Emanuels et al. [15]). In particular, it has been found that children exposed to poor parenting, such as abuse and low warmth and support, are at greater risk for DV [35]. Tyler et al. [36], using data from the National Longitudinal Study of Adolescent Health, a nationally representative dataset of adolescents in the United States, also found that low parental warmth was associated with the perpetration of DV. Specifically, maternal warmth, as well as paternal and maternal hostility, were significant predictors of DV [37].

### 1.2. Moral Disengagement and Youth Violence

Moral disengagement is a cognitive variable that is receiving more attention in studies examining violent behavior. More specifically, moral disengagement has been related to aggressive behavior in childhood and adolescence [38], particularly among individuals aged between 12 and 18 years old (review by Gini et al. [39]). Moral disengagement, addressed by Bandura within his social cognitive theory, explains how individuals can justify harmful behaviors without feeling guilt [40]. Bandura introduced the concept of a self-regulatory process of internal moral control, whereby individuals regulate their behavior according to moral standards acquired during their socialization [41,42]. Moral behavior, according to Bandura, is neither static nor automatic but results from the activation or deactivation of self-regulatory processes, which are subject to the influence of cognitive, affective, and social factors. This explains why individuals with similar principles may act in different ways. Within this framework, moral disengagement is understood as a process that allows moral self-control to be deactivated through eight mechanisms: (1) Moral justification: reinterpreting a harmful action as morally acceptable; (2) Euphemistic language: using neutral terms when describing immoral acts to reduce their negative impact; (3) Advantageous comparison: minimizing the seriousness of an act by contrasting it with a worse one; (4) Diffusion of responsibility: diluting responsibility for one’s own behavior by sharing it with a group; (5) Displacement of responsibility: attributing blame to an authority figure or external circumstances; (6) Distortion of consequences: minimizing, denying or ignoring the harm caused; (7) Dehumanization: perceiving the victims of one’s actions as lacking in human qualities, which facilitates the justification of harmful actions towards them; (8) Attribution of blame: holding the victim responsible for what happened, justifying the harm caused towards them. These eight mechanisms are organized into four strategies: the first three are grouped into the cognitive reconstruction strategy, the fourth and fifth mechanisms are grouped into the obscuring personal responsibility strategy, the sixth mechanism is part of the minimizing consequences strategy, and the last two mechanisms are grouped into the blaming the victim strategy [41,42]. In the scientific literature, moral disengagement has been analyzed by specific mechanisms, by strategies, and in a global manner. However, studying the eight mechanisms of moral disengagement rather than grouping them by strategies or globally allows us to identify more precisely which factors contribute to immoral behavior in specific situations since these mechanisms do not necessarily uniformly activate in all contexts.

In the field of CPV, moral disengagement has been scarcely studied. A study involving Spanish adolescents from a community sample finds that moral disengagement is positively related to CPV toward both parents [43]. Contradictory results are found when this variable is further analyzed by strategies. Espejo-Siles et al. [44], also studying Spanish adolescents, analyzed three of the four moral disengagement strategies: concretely, cognitive reconstruction, minimizing consequences, and blaming the victim. They found that none of these strategies predicted violence against parents. However, Baustista-Aranda et al. [28] found that precisely these three moral disengagement strategies were significant predictors of CPV, with cognitive reconstruction being the strategy that played the most relevant role. This study suggests that, in the context of CPV, this moral disengagement strategy is the most effective psychological mechanism for the disengagement of moral self-sanctions compared to other moral disengagement strategies. At this point, it seems necessary to know the role played by the different mechanisms of moral disengagement in CPV, which could be useful both to clarify some previous inconsistencies and to further deepen the role of moral disengagement.

In the context of PV, it has been common to analyze the construct of moral disengagement through the eight mechanisms that comprise it. Although individuals who bully others may recognize that such behaviors are morally wrong [45], they may use moral disengagement mechanisms to justify their actions despite violating their moral standards [46]. Research reveals a consistent association between moral disengagement and PV [32,39,46,47]. One finding that is consistent across studies is the predictive role of cognitive reconstruction strategy in PV [44,48], more precisely, the moral justification mechanism [49,50]. This mechanism has been identified as the only significant moral disengagement mechanism in some studies [44,49] and the most relevant in others [48]. Other moral disengagement mechanisms that show less consensus but also predict PV are euphemistic language, displacement of responsibility [51], attribution of blame [50], dehumanization, and distortion of consequences [48].

Concerning DV, few studies have examined the relationship between moral disengagement and this type of violence. One study involving university students found that moral disengagement is a significant predictor of DV [52] and, more specifically, the obscuring personal responsibility strategy [38]. Along the same lines, emotional DV has been related to the “rationalization dimension” (which groups together the mechanisms of moral justification, euphemistic language, and distortion of consequences) [38], and physical DV has been associated with the mechanism of diffusion of responsibility [53], while the mechanisms of justification of violence and dehumanization are not significantly related to DV [54].

### 1.3. Parental Warmth, Moral Disengagement, and Youth Violence

The relationship between the lack of parental warmth and youth violent behaviors may be influenced by several intermediary factors, as found in the CPV field. In particular, the lack of perceived parental warmth can lead to psychological maladjustment in children, manifesting in cognitive, emotional, and social difficulties, which subsequently contribute to the development of CPV [55]. One of the intermediary factors that could be influencing the relationship between the lack of parental warmth and youth violence is the moral disengagement mechanism. For example, a recent study has highlighted that moral disengagement mechanisms (moral justification and euphemistic language in particular) mediate the association between harsh parenting and adolescent aggression in general [56]. To advance understanding of the relationship between parental warmth, moral disengagement, and different types of youth violence, it is essential to address several gaps in the literature.

In the context of CPV, previous research [28] has explored whether the relationship between the lack of parental warmth and CPV is mediated by moral disengagement strategies, but the specific moral disengagement mechanisms involved remain unclear. In the mentioned research [28], cognitive reconstruction was identified as the key moral disengagement strategy mediating the relationship between paternal and maternal warmth-communication and CPV, as well as in the relationship between paternal and maternal criticism-rejection and CPV. In other words, “through cognitive reinterpretation of their violent acts, adolescents justify and legitimize their actions, making them acceptable and thus avoiding guilt, distress, or remorse” [28] (p 12).

In the field of PV, previous studies have explored whether the relationship between certain parental practices and PV is mediated by moral disengagement, but these relationships have not been examined with lack of parental warmth as a predictor variable nor with specific moral disengagement mechanisms as mediators. These studies found that moral disengagement mediates the relationship between harsh parental discipline (psychological aggression, corporal punishment, and physical abuse) and PV [57], as well as between parental monitoring and cyberbullying behaviors [58].

Lastly, in the context of DV, the relationship between lack of parental warmth and this type of violence through the effect of moral disengagement remains unexplored, even less through the moral disengagement mechanisms.

This study aims to fill these gaps by identifying the specific mechanisms of moral disengagement that mediate the relationship between the lack of parental warmth and the different forms of youth violence reviewed.

### 1.4. Purpose of the Present Study

Objective 1. To analyze the relationship between youth violence (CPV, PV, and DV) and (1) perceived parental warmth (both warmth-communication and criticism-rejection) and (2) moral disengagement mechanisms (moral justification, euphemistic language, advantageous comparison, displacement of responsibility, diffusion of responsibility, distortion of consequences, dehumanization and attribution of blame).

**H1.**  *CPV [14,28], PV [32], and DV [37] are expected to be negatively related to parental warmth-communication and positively related to parental criticism-rejection.*

**H2.**  *In turn, moral disengagement mechanisms will be positively related to CPV [28,43], PV [32], and DV [52].*

Objective 2. To explore the mediating role of moral disengagement mechanisms in the relationship between the lack of perceived parental warmth and CPV, PV, and DV, and to determine if these mechanisms differ according to the type of youth violence.

**H3.**  *Moral justification, euphemistic language, and/or advantageous comparison moral disengagement mechanisms mediate the relationship between the lack of perceived parental warmth and CPV [28].*

**H4.**  *Moral disengagement will mediate the relationship between the lack of perceived parental warmth and PV [57,58]. No specific hypotheses are proposed for the specific mechanisms of moral disengagement involved in this relationship, nor for DV, given the absence of previous evidence.*

## 2. Materials and Methods

### 2.1. Participants

The sample consisted of 2124 Spanish adolescents (57.9% girls) aged between 13 and 17 years (*M*_age_ = 14.9, *SD* = 1.3). Participants were recruited from 25 schools located in various Spanish regions: 34.3% from the south (Córdoba, Granada, Málaga, and Cádiz), 60.5% from the centre (Ciudad Real), and the remaining 5.2% from the north of Spain (Oviedo). The majority of the participants lived with both parents (82%), while the remaining participants lived with the mother (6.5%), with the father (0.8%), or in reconstituted families (10.7%). 99.3% of the sample were biological children, and most of the participants’ parents were married (78.9%). The minimum sample size required at a 99% confidence level was 1841 participants. To determine the sample size, the total population of adolescents in Spain aged 13–17 years was considered.

### 2.2. Instruments

Child-to-Parent Violence Questionnaire, adolescent version (CPV-Q) [59]. It assesses the frequency of different violent behaviors (psychological, physical, financial, and control/domain) directed towards parents (α = 0.83) during the last year through 14 parallel items (father and mother, e.g., “I have told my parents that at home they have to do what I want”) answered on a 5-point Likert scale (0 = never to 4 = very often, six times or more).

Violence in School and Leisure Assessment Questionnaire (C.E.V.E.O. questionnaire) [60]. It assesses the frequency of different behaviors of violence towards peers over the last two months in the school context and in leisure situations. It contains 28 items (α = 0.87), e.g., “Threatening him/her in order to scare him/her”, answered on a 4-point Likert scale (1 = never to 4 = very much).

Dating Violence Questionnaire for Victimization and Perpetration (DVQ-VP) [61]. It assesses the frequency of different violent behaviors (coercion, sexual violence, physical violence, detachment, and humiliation) towards a partner through 20 items (α = 0.79), e.g., “You hurt him/her with some object”, answered on a 5-point Likert scale (0 = never to 4 = almost always).

Warmth Scale, child’s version (W-S) [62]. This instrument comprises two factors: the warmth-communication scale (α = 0.94) and the criticism-rejection scale (α = 0.92), both composed of 10 parallel items (for father and mother, e.g., “I feel that I am a bother to him/her”) answered on a 5-point Likert scale (0 = never to 4 = always).

Mechanisms of Moral Disengagement Scale (MMDS-S) [63], Spanish validation [64]. It assesses the tendency to use eight moral disengagement mechanisms (moral justification, euphemistic language, advantageous comparison, displacement of responsibility, diffusion of responsibility, distortion of consequences, dehumanization, and attribution of blame) through 32 items (α = 0.85), e.g., “Damaging some property is no big deal when you consider that others are beating people up”, answered on a 5-point Likert scale. (1 = strongly disagree to 5 = strongly agree).

### 2.3. Procedure

This cross-sectional study employs a survey population design [65]. The research received a favorable report from the Ethics Committee of the University of Jaén (reference: OCT.19/1.PRY). Authorizations were obtained from the Public Administration in the field of education and from the educational centers. Subsequently, signed informed consent was obtained from the parents of underage adolescents, as well as from the adolescents themselves. Participation was voluntary and involved completing a battery of confidential questionnaires administered by trained researchers, conducted in classrooms in person and in groups. Each adolescent was seated at an individual table and was assigned an anonymous identification code. No incentives were offered for participation in the study.

### 2.4. Data Analysis

**Figure 1:**
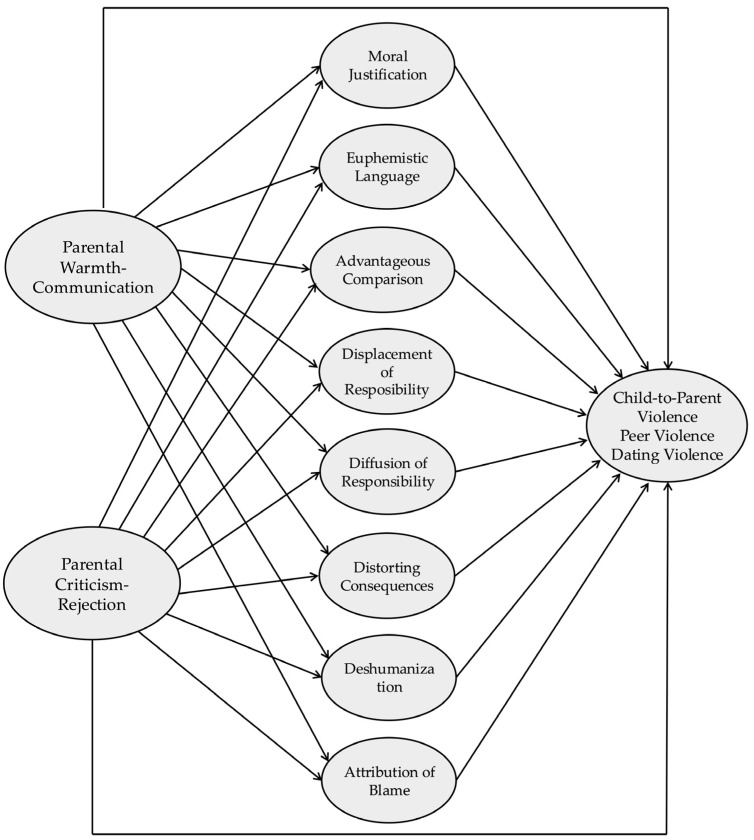
Proposed Mediation Theoretical Model.

A significance level of *p* < 0.05 was established for all analyses. Correlational analyses were conducted initially to examine associations among different types of youth violence, perceived parental warmth, and moral disengagement mechanisms. Subsequently, mediational analyses were conducted to examine the direct and indirect relationships outlined in the theoretical model proposed in Figure 1.

The correlation assumptions between the predictor, mediator, and dependent variables were verified. Variables that did not meet these criteria (diffusion of responsibility) were excluded from the model. All other assumptions were satisfied, except for normality, a frequent condition in studies on violence in the general population. One model was fitted for each type of youth violence. Specifically, we examined (1) the direct effect of perceived parental warmth (predictor variables: warmth-communication and criticism-rejection) on the prediction of youth violence (dependent variables: CPV, PV, and DV) and (2) the indirect effect of moral disengagement mechanisms (mediator variables: moral justification; euphemistic language; advantageous comparison; displacement of responsibility; distorting consequences; dehumanization and attribution of blame) on the relationship between perceived parental warmth and the types of youth violence. The mediation model was estimated in R software version 4.4.2 [66] using the *r* lavaan package [67].

**Figure 2:**
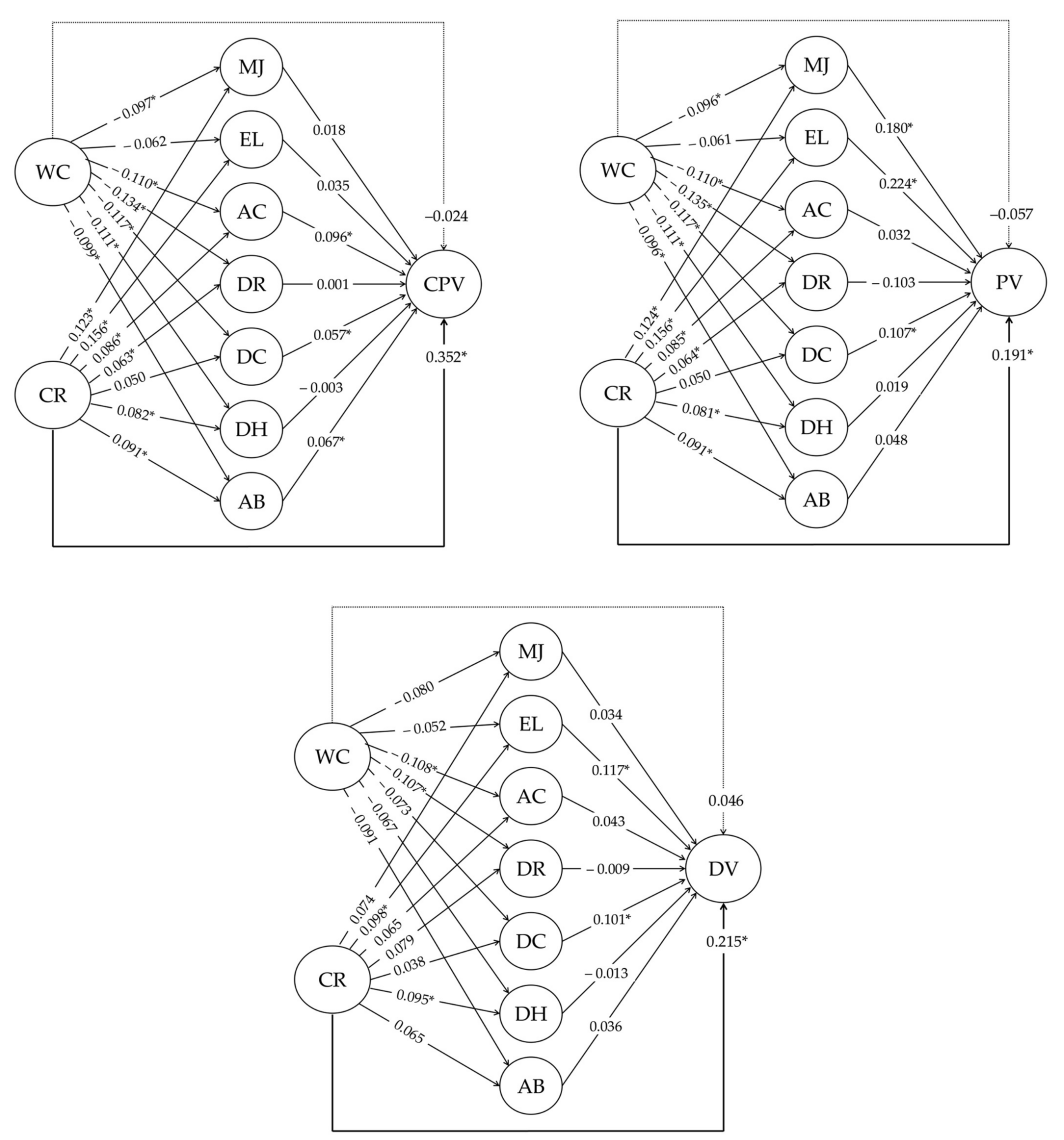
Results of The Proposed Mediational Models. WC = parental warmth-communication; CR = parental criticism-rejection; MJ = moral justification; EL = euphemistic language; AC = advantageous comparison; DR = displacement of responsibility; DC = distorting consequences; DH = dehumanization; AB = attribution of blame; CPV = child-to-parent violence; PV = peer violence; DV = dating violence. * Statistically significant relationships.

Given the absence of normality, nonparametric bootstrapping was implemented with 10,000 resamples, applying the BCa (bias-corrected and accelerated bootstrap) method to calculate the 95% confidence intervals of the indirect effects [68], which are considered significant if they do not include the value 0 [69]. Additionally, since Z-based *p*-values assume normality, the significance of indirect effects was also evaluated with the *r* robmed package [70], which generates *p*-values from bootstrap replicates, providing greater robustness against non-normal distributions [70,71,72]. Figure 2 shows the standardized coefficients of the model components and the total effect of the relationship between the independent and dependent variables through the mediators.

## 3. Results

Table 1 shows that CPV, PV, and DV are negatively associated with parental warmth-communication (*r* = −0.24, *r* = −0.17, *r* = −0.10, respective) and positively associated with parental criticism-rejection (*r* = 0.36, *r* = 0.22, *r* = 0.19, respective), with CPV exhibiting the strongest relationship with these variables. Additionally, all three types of youth violence are positively related to all moral disengagement mechanisms except for diffusion of responsibility. In particular, moral justification, euphemistic language, advantageous comparison, displacement of responsibility, distortion of consequences, dehumanization, and attribution of blame are associated with CPV (*r* = 0.07 to *r* = 0.22), PV (*r* = 0.10 to *r* = 0.36) and DV (*r* = 0.12 to *r* = 0.23), with PV showing the strongest relationship with these variables.

The proposed mediation theoretical model was tested (see Figure 2) for each type of youth violence (CPV, PV, and DV). The results indicate that the parental warmth-communication variable does not predict any type of youth violence, whereas parental criticism-rejection is a significant predictor of all three types of youth violence (CPV, PV, and DV), especially CPV. As for the mechanisms of moral disengagement, some of them significantly mediate the relationship between high levels of parental criticism-rejection and youth violence, which also vary according to the type of violence (see Table 2).

CPV model: Parental criticism-rejection is directly related to CPV (β = 0.327, 95% CI [0.173, 0.261]) and is even more strongly related indirectly (β = 0.352, 95% CI [0.190, 0.278]) through the effects of advantageous comparison (β = 0.008, 95% CI [0.001, 0.013]) and attribution of blame (β = 0.006, 95% CI [0.001, 0.011]). Parental warmth-communication is neither directly (β = 0.004, 95% CI [−0.026, 0.030]) nor indirectly (β = −0.024, 95% CI [−0.040, 0.016]) related to CPV.

PV model: As in the previous model, parental criticism-rejection is directly related to PV (β = 0.135, 95% CI [0.037, 0.103]) and is indirectly related more strongly (β = 191, 95% CI [0.063, 0.133]) through the effects of moral justification (β = 0.013, 95% CI [0.003, 0.013]), euphemistic language (β = 0.035, 95 % CI [0.010, 0.028]), and distortion of consequences (β = 0.005, 95 % CI [0.000, 0.008]). Again, parental warmth-communication is neither directly (β = −0.024, 95 % CI [−030, 0.016]) nor indirectly (β = −0.057, 95 % CI [−0.044, 0.005]) related to PV.

DV model: Parental criticism-rejection is directly (β = 193, 95% CI [0.035, 0.100]) and indirectly (β = 0.215, 95% CI [0.041, 0.110]) related to DV. However, none of the mediators reach conventional statistical significance (*p* > 0.05). Nevertheless, the confidence intervals associated with the indirect effect of euphemistic language do not include the value 0 (β = 0.012, 95% CI [0.000, 0.019]), and the *p*-value approaches statistical significance (*p* = 0.052). Also, in this model, there is no direct (β = 0.068, 95% CI [−0.003, 0.041]) or indirect relationship (β = 0.046, 95% CI [−0.010, 0.035]) between parental warmth-communication and DV.

## 4. Discussion

From an integrated approach, the purpose of this study was to analyze the role of perceived parental warmth and the moral disengagement mechanisms, as well as the interconnection between these variables, in child-to-parent violence (CPV), peer violence (PV), and dating violence (DV), and to compare whether they play a differential role depending on the type of youth violence. The first objective was to analyze the relationship between youth violence (CPV, PV, and DV) and (1) perceived parental warmth and (2) moral disengagement mechanisms.

### 4.1. Parental Warmth and Youth Violence

Regarding perceived parental warmth, in line with expectations from Hypothesis 1, we found that CPV, PV, and DV were negatively related to parental warmth-communication and positively related to parental criticism-rejection. These findings provide additional evidence to support previous studies indicating that lower perceived parental warmth-communication and higher perceived parental criticism-rejection are associated with higher levels of youth violence [14,28,32,37]. On the other hand, although numerous parental practices are linked to violent behavior, the specific role of perceived parental warmth-communication and criticism-rejection has been little researched in some types of youth violence, for example, PV and DV. Therefore, the findings of this study also broaden the range of dysfunctional parenting practices related to the three types of youth violence analyzed.

More precisely, the results of the mediational models indicate that high levels of parental criticism-rejection significantly predict CPV, PV, and DV. This finding is consistent with previous research in the field of CPV [20,24,28,55] and clarifies the predictive role that these particular parental practices play in PV [34] and DV [37], which have been less studied. Previous research has found that low levels of warmth-communication also predict CPV [28,59]. However, our study shows that, when considering them simultaneously, only criticism-rejection maintains a significant effect. This finding suggests that it is the active presence of negative interactions, such as criticism and rejection, that has a more direct impact on the manifestation of CPV. Thus, our study further clarifies the role of both dimensions of the perceived lack of parental warmth by suggesting that criticism-rejection is the key factor when analyzed jointly with low warmth-communication. More importantly, given that these results are replicated for the three types of youth violence, the study suggests that this dimension of the lack of perceived parental warmth is a common risk factor for CPV, PV, and DV. This confirms that adolescents who feel rejected by their parents are at greater risk of engaging in violent behavior, not only in one specific context but across three different contexts, thereby tripling the likelihood of generalized violence. Promoting affectionate parental dynamics could serve as a protective factor against violence in different interpersonal contexts. Considering that several studies have pointed out the interrelation among these three types of violence [13] and their tendency to co-occur [10,11,12], promoting parental warmth may even prevent the generalization of violence to other contexts. This idea would reinforce Ibabe and Bentler’s [29] suggestion that affectivity in family relationships is the most important aspect of preventing violence.

By type of violence, the results suggest that high levels of criticism-rejection play a more significant role in the development of CPV than PV and DV. Given that CPV, unlike the other two types of violence, occurs in the family context and is directed toward parents, it is expected that the lack of parental warmth would be a primary factor in its development compared to the other types of violence. This idea is consistent with previous studies indicating that a lack of perceived parental warmth is one of the key variables that differentiate the offenders who have committed CPV crimes from those offenders who have committed other crimes [25,26]. The findings highlight the necessity for greater social awareness regarding the importance of warmth-based parenting in preventing youth violence in general, especially CPV. This perspective is supported by studies indicating that parental warmth is an essential variable in children’s psychosocial adjustment [18] and also exerts a protective role in the development of youth violence [24,31].

### 4.2. Moral Disengagement and Youth Violence

Regarding moral disengagement mechanisms, a positive relationship was expected with CPV [28,43], PV [32], and DV [52]. The results partially confirm Hypothesis 2. All moral disengagement mechanisms are positively and significantly related to all three types of violence, except for diffusion of responsibility. These results align with prior research indicating that higher levels of moral disengagement are associated with increased levels of CPV [43], PV [32,39,46,47], and DV [52], and more precisely, certain moral disengagement strategies with CPV [28], PV [44,48], and DV [38]. However, the specific relationship between the different mechanisms that comprise moral disengagement and youth violence has primarily been analyzed concerning PV [49,50,51]. The findings of the present study extend this understanding by demonstrating that nearly all moral disengagement mechanisms are related to all these types of violence, suggesting a shared presence of dysfunctional social-cognitive processing in youth violence in general.

### 4.3. Parental Warmth, Moral Disengagement, and Youth Violence

The second aim of this study was to explore the mediating role of moral disengagement mechanisms in the relationship between the lack of perceived parental warmth and youth violence (CPV, PV, and DV). The results indicate that the relationship between the lack of perceived parental warmth (criticism-rejection dimension) and the three types of youth violence is mediated by moral disengagement mechanisms, confirming our hypotheses 3 [28] and 4 [57,58]. This aligns with the Wang et al. [56] study in which moral disengagement mediated the association between harsh parenting and adolescent aggression in general. Moral socialization begins within the family environment, making family relationships crucial in the process of learning and internalizing moral principles about right and wrong. It appears that parental criticism-rejection contributes to the development of moral disengagement mechanisms in adolescents, and this disengagement may facilitate adolescents’ implication in interpersonal violence. This suggests that families lacking a solid emotional bond may foster beliefs that shape dysfunctional cognitive mechanisms in adolescents. Indeed, it has been highlighted that the lack of perceived parental warmth impacts adolescents’ cognitive, emotional, and social functioning, which, in turn, leads to CPV [55]. Regarding the role of parental warmth-communication, previous studies suggest that this dimension of the lack of parental warmth is a risk factor for problems in sociocognitive processing that are specifically related to parents; that is, children with low levels of parental warmth-communication tend to interpret their parents’ intentions in a hostile manner, which in turn increases the likelihood of exercising CPV [55]. The present study indicates that low levels of parental warmth-communication do not operate through the cognitive mechanisms of moral disengagement in the development of CPV, perhaps because they do not focus on the relationship with parents in particular. Neither are they implicated in the development of PV or DV, so it may be that the lack of warmth-communication generates other types of emotional, social, and even cognitive deficits, but not necessarily deficits in moral self-control.

Concerning the differential role of moral disengagement mechanisms, the present study offers novel information by revealing that most of the moral disengagement mechanisms involved in the relationship between the lack of parental warmth and youth violence vary by type of violence. This suggests that CPV, PV, and DV may be characterized by different cognitive mechanisms associated with the lack of perceived parental warmth.

#### 4.3.1. Child-to-Parent Violence

Bautista-Aranda et al. [28] analyzed the role of four moral disengagement strategies in the relationship between the lack of parental warmth during childhood and CPV, finding that the cognitive reconstruction strategy (moral justification, euphemistic language, and advantageous comparison mechanisms) significantly mediated this relationship. The present study shows that the lack of parental warmth during the last year is specifically related to the advantageous comparison mechanism, which is linked to CPV. Through advantageous comparison, which emerges as a specific mediator for CPV, adolescents compare their harmful behavior with behavior that is considered even more harmful so that the adolescent’s behavior is perceived as less serious or even benevolent [42]. In this sense, adolescents may compare the violence they exert towards their parents with the poor affection they perceive from them, and through the advantageous comparison, they could see their behavior as not as harmful compared to that of their parents. Additionally, attribution of blame to the victim is also a specific mediator of the relationship between the lack of parental warmth and CPV in this study, contrasting with the findings of Bautista-Aranda et al. [28]. This cognitive mechanism may become more pronounced during adolescence, which could explain why in Bautista-Aranda et al. [28] research the attribution of blame is not related to the lack of parental warmth that adolescents perceived before the age of 10. Through this mechanism, adolescents blame their victims for their harmful behavior, which is considered a defensive reaction to the suffering that the victim is causing them [42]. Therefore, adolescents who do not perceive the warmth of their parents would tend to self-excuse the violence exercised towards them for reactive reasons or because they consider their own violent actions as a response to the circumstances (whether real or imagined). Supporting this notion, the previously mentioned study [55] found that the attribution of hostile intentions to parental behavior mediates the relationship between the lack of parental warmth and CPV motivated by reactive reasons.

#### 4.3.2. Peer Violence

Concerning the cognitive mechanisms involved in the relationship between the lack of perceived parental warmth and PV, Bartolo et al. [58] and Fan et al. [57] found that moral disengagement mediated the relationship between some parental practices and PV behaviors. The findings of this study reveal that the concrete mechanisms that mediate the relationship between the lack of perceived parental warmth and PV are moral justification, euphemistic language, and distortion of consequences. The moral justification mechanism, a specific mediator of PV, involves harmful behavior becoming socially acceptable because it fulfills a valued social or moral purpose, such as protecting honor or reputation [42]. Adolescents may attempt to compensate for affective deprivation at home by seeking group acceptance or affiliation with certain peer groups where violence is socially accepted so that the moral justification associated with lack of parental warmth would increase the risk of adolescents engaging in PV. In the distortion of consequences mechanism, which is also a specific mediator of PV, harmful behaviors that are performed for self-benefit or social incentives are minimized or ignored as the benefits of such behaviors are easily recalled [42]. When adolescents perceive a lack of parental warmth, they may seek social rewards through PV. By minimizing the negative effects of violence, adolescents may become disinhibited from engaging in such violent behaviors.

#### 4.3.3. Dating Violence

Euphemistic language stands out in this study as the only mechanism of moral disengagement that could be facilitating the already established relationship between the perceived lack of parental warmth and DV. The effect of this mediator did not reach conventional statistical significance, although it did reach that determined by CI. The absence of previous evidence in this area suggests the need for further research. In particular, it would be convenient to analyze the effect of this mechanism in larger samples than the one considered in the present study (*N* = 2124) since only 799 of the total sample were in a couple relationship. Consequently, the effect of euphemistic language likely reaches conventional statistical significance in larger samples in the same way that it reaches the statistical significance of the CI generated from 10,000 interactions in this study. Pending future studies to confirm the above, euphemistic language would stand out in this study as a common cognitive mechanism implicated in the relationship between the perceived lack of parental warmth and two of the three types of youth violence analyzed. This could suggest that adolescents who perceive a lack of parental warmth tend to develop patterns of thinking characterized by language that conveniently masks harmful behavior as benign [42], which, in turn, increases the risk of generalized youth violence. In this sense, it would be especially important to pay attention to the language used by adolescents and to incorporate the modification of cognitive patterns linked to the use of euphemistic labels in intervention programs for youth violence.

In summary, the present study shows that the lack of perceived parental warmth, specifically the high levels of parental criticism-rejection, is a common risk factor for CPV, PV, and DV, although it seems to play a more relevant role in the development of CPV. More importantly, the lack of perceived parental warmth is related to CPV, PV, and DV through moral disengagement mechanisms, meaning that the risk of perceived lack of parental warmth is even higher in the presence of these moral disengagement mechanisms. Furthermore, highlights a differential contribution of moral disengagement mechanisms in this relationship, suggesting that the lack of parental warmth influences each of these types of violence through different cognitive mechanisms. Particularly, adolescents who experience parental criticism-rejection are more likely to (1) compare their harmful behavior to actions considered even more harmful and blame the victim for their harmful behavior when committing violence towards parents; (2) justify the harmful behavior as serving a socially valued purpose and minimize or ignore the harm by recalling the benefits or social incentives of the harmful behavior when committing violence towards peers; (3) using euphemistic language that masks and transforms harmful behavior into good behavior when committing violence toward peers and partners.

### 4.4. Limitations, Contributions, and Future Research

A cross-sectional design was chosen in this study because it allows us to identify patterns of relationship between variables and to establish a basis for future longitudinal research. However, one limitation of this approach is the impossibility of establishing causality and assessing temporal changes. In this sense, future longitudinal studies could provide more precise information on the relationships studied. Additionally, given that the sample is limited to six Spanish geographic regions and the type of sampling was not random, the generalizability of the results to other regions and countries is limited. Greater geographic diversity and stratified random sampling are needed to increase the representativeness of the results. Finally, the findings are based on self-reported data from adolescents. Biases associated with social desirability could have influenced participants’ responses, although the voluntary and anonymous nature of the responses could have counteracted social desirability. To mitigate these limitations in future research, we suggest the use of complementary approaches, such as the incorporation of observational or third-party reported data, such as the perceptions of parents, peers, and partners of participants.

Despite its limitations, this study contributes to a better understanding of CPV, PV, and DV, offering several practical implications. This study suggests the importance of implementing youth violence prevention programs that involve parents to promote and strengthen parenting warmth-focused practices. These practices could include more effective and respectful communication with children and more effective learning skills of expressing support and affection for them, as well as reducing criticism and making it constructive, avoiding constant disapproval and expressions of hostility. Specifically, interventions could focus on (1) fostering emotional connection and moral engagement by training parents in active listening, emotional validation, and modeling empathy to minimize patterns of moral disengagement, such as displacement of responsibility, and (2) reducing punitive behaviors through the promotion of positive parenting to decrease, for example, adolescent justification of violence. Additionally, it would be essential in the intervention with children to address the euphemistic label-based thinking pattern of adolescents involved in youth violence and to direct more resources toward modifying dysfunctional beliefs and attitudes linked to the particular type of violence being addressed.

The results of this study also suggest several important directions for future research. It would be necessary to further investigate the mechanisms of moral disengagement that mediate the relationship between the perceived lack of parental warmth and the types of youth violence separately. Understanding the nuances of these cognitive processes could enhance our comprehension of how different types of violence are justified and perpetuated. One approach could be to analyze the motivations for different types of youth violence. For example, Cano-Lozano et al. [55] found that the lack of parental warmth was related to different dysfunctional components of sociocognitive processing, and these varied depending on whether the CPV was motivated by reactive or instrumental reasons. Additionally, it would be interesting to examine the role of other possible mediators and moderators in this relationship and to complement mediational analyses with regression models. This approach may provide a more comprehensive understanding of the psychological mechanisms involved in the development of CPV, PV, and DV. On the other hand, this study focuses on the Spanish context, so it is important to consider the cross-cultural perspective. Dimensions of parental warmth may vary significantly across cultures due to differences in social norms, parenting practices, and family values. Extensive cross-cultural research in the IPAR theory [17] concludes that the expression of parental acceptance-rejection should be analyzed in the cultural context where it is manifested. Also, moral disengagement could be influenced by cultural factors that modulate the perception of social norms and values depending on levels of social tolerance to violence, exposure to armed conflict, the degree to which educational systems reinforce ethical and prosocial norms, etc. This could have implications for how parental warmth and moral disengagement relate to youth violence in different cultures. Thus, future studies could explore the dynamics analyzed in samples from different countries, comparing the relational patterns observed in different cultural contexts. This would not only enhance the global understanding of these phenomena but would also allow the design of more effective interventions.

## 5. Conclusions

In conclusion, this study suggests that parental criticism-rejection, considered part of the negative end of the lack of parental warmth construct, not only increases the risk of youth violence (child-to-parent violence, peer violence, and dating violence) but is also related to the moral disengagement mechanisms that legitimize harmful behavior, which in turn could increase these types of youth violence. More importantly, it highlights the existence of a differential contribution of moral disengagement mechanisms in the relationship between the lack of perceived parental warmth and the types of youth violence. These findings suggest that adolescents exposed to the same risk factor may be more likely to engage in different types of youth violence through the development of different dysfunctional cognitive mechanisms. Consequently, it would be important to promote parenting practices based on warmth, as well as to prevent or modify the cognitive mechanisms underlying youth violence, with special attention to those specifically related to each type of violence.

## Figures and Tables

**Table 1:** Correlations Between Study Variables.

		1	2	3	4	5	6	7	8	9	10	11	12	13
1	CPV	-												
2	PV	0.41 ***	-											
3	DV	0.27 ***	0.39 ***	-										
4	WC	−0.24 ***	−0.17 ***	−0.10 **	-									
5	CR	0.36 ***	0.22 ***	0.19 ***	−0.65 ***	-								
6	MJ	0.19 ***	0.30 ***	0.19 ***	−0.18 ***	0.19 ***	-							
7	EL	0.20 ***	0.36 ***	0.23 ***	−0.16 ***	0.20 ***	0.50 ***	-						
8	AC	0.22 ***	0.25 ***	0.19 ***	−0.17 ***	0.16 ***	0.46 ***	0.48 ***	-					
9	DR	0.14 ***	0.10 ***	0.12 ***	−0.17 ***	0.15 ***	0.32 ***	0.28 ***	0.37 ***	-				
10	DF	0.07 **	0.02	−0.05	−0.08 ***	0.04	0.30 ***	0.18 ***	0.30 ***	0.37 ***	-			
11	DC	0.19 ***	0.28 ***	0.19 ***	−0.15 ***	0.13 ***	0.46 ***	0.46 ***	0.47 ***	0.39 ***	0.26 ***	-		
12	DH	0.15 ***	0.21 ***	0.14 ***	−0.17 ***	0.15 ***	0.38 ***	0.37 ***	0.37 ***	0.26 ***	0.25 ***	0.35 ***	-	
13	AB	0.21 ***	0.25 ***	0.20 ***	−0.16 ***	0.15 ***	0.51 ***	0.44 ***	0.46 ***	0.41 ***	0.29 ***	0.44 ***	0.49 ***	
	*M*	10.82	34.56	3.51	56.81	14.32	9.39	6.04	7.39	8.79	11.75	7.63	7.52	8.12
	*SD*	8.46	6.54	4.68	16.88	12.50	3.43	2.26	2.65	3.00	3.65	2.80	2.90	2.87

*N* = 2124; CPV = child-to-parent violence; PV = peer violence; DV = dating violence; WC = parental warmth-communication; CR = parental criticism-rejection; MJ = moral justification; EL = euphemistic language; AC = advantageous comparison; DR = displacement of responsibility; DF = diffusion of responsibility; DC = distorting consequences; DH = dehumanization; AB = attribution of blame; *M* = mean; *SD* = standard deviation. ** *p* < 0.01. *** *p* < 0.001.

**Table 2:** Results of The Mediational Models for Child-to-Parent Violence, Peer Violence and Dating Violence.

		Child-to-Parent Violence Model ^1^							Peer Violence Model ^2^							Dating Violence Model ^3^						
Efects	Path	β	*SE*	LLCI	ULCI	*z*	*p*	*p_Boot_*	β	*SE*	LLCI	ULCI	*z*	*p*	*p_Boot_*	β	*SE*	LLCI	ULCI	*z*	*p*	*p_Boot_*
Indirect	WC→MJ→YV	−0.002	0.002	−0.004	0.002	−0.555	0.579	0.539	−0.010	0.002	−0.008	−0.001	−2.374	0.018	0.001	−0.003	0.001	−0.005	0.001	−0.489	0.625	0.562
	WC→EL→YV	−0.002	0.001	−0.005	0.001	−0.888	0.375	0.309	−0.014	0.003	−0.011	0.000	−1.919	0.055	0.038	−0.006	0.002	−0.008	0.000	−0.879	0.379	0.323
	WC→AC→YV	−0.011	0.002	−0.011	−0.002	−2.169	0.030	0.002	−0.004	0.001	−0.005	0.001	−0.986	0.324	0.268	−0.005	0.001	−0.005	0.001	−0.849	0.396	0.315
	WC→DR→YV	0.000	0.002	−0.004	0.003	−0.023	0.982	0.993	0.014	0.002	0.002	0.009	2.969	0.003	0.000	0.001	0.001	−0.002	0.003	0.198	0.843	0.830
	WC→DC→YV	−0.007	0.002	−0.008	0.000	−1.769	0.077	0.042	−0.013	0.002	−0.009	−0.002	−2.628	0.009	0.000	−0.007	0.002	−0.007	0.000	−1.132	0.258	0.161
	WC→DH→YV	0.000	0.001	−0.003	0.003	0.101	0.920	0.913	−0.002	0.001	−0.003	0.001	−0.679	0.497	0.468	0.001	0.001	−0.001	0.003	0.254	0.800	0.754
	WC→AB→YV	−0.007	0.002	−0.008	0.000	−1.694	0.090	0.037	−0.005	0.001	−0.005	0.000	−1.423	0.155	0.079	−0.003	0.001	−0.005	0.002	−0.556	0.578	0.511
	CR→MJ→YV	0.002	0.003	−0.003	0.007	0.574	0.566	0.539	0.013	0.002	0.003	0.013	2.680	0.007	0.000	0.003	0.002	−0.001	0.006	0.490	0.624	0.584
	CR→EL→YV	0.006	0.004	−0.002	0.012	1.004	0.315	0.282	0.035	0.005	0.010	0.028	3.768	0.000	0.000	0.012	0.004	0.000	0.019	1.081	0.280	0.052
	CR→AC→YV	0.008	0.001	0.001	0.013	1.985	0.047	0.010	0.003	0.001	−0.001	0.005	0.990	0.322	0.276	0.003	0.001	−0.001	0.005	0.704	0.482	0.417
	CR→DR→YV	0.000	0.001	−0.002	0.003	0.021	0.983	0.987	−0.007	0.002	−0.008	0.000	−1.731	0.083	0.116	−0.001	0.001	−0.003	0.002	−0.187	0.852	0.858
	CR→DC→YV	0.003	0.002	0.001	0.007	1.114	0.265	0.144	0.005	0.002	0.000	0.008	1.386	0.166	0.041	0.004	0.002	−0.001	0.007	0.666	0.505	0.439
	CR→DH→YV	0.000	0.001	−0.003	0.003	−0.098	0.922	0.915	0.002	0.001	−0.001	0.004	0.661	0.508	0.470	−0.001	0.001	−0.004	0.002	−0.285	0.776	0.749
	CR→AB→YV	0.006	0.003	0.001	0.011	1.599	0.110	0.040	0.004	0.002	0.000	0.006	1.441	0.150	0.080	0.002	0.002	−0.001	0.005	0.515	0.607	0.570
Direct	WC→YV	0.004	0.014	−0.026	0.030	0.128	0.898	0.883	−0.024	0.012	−0.030	0.016	−0.744	0.457	0.450	0.068	0.011	−0.003	0.041	1.581	0.114	0.127
	CR→YV	0.327	0.022	0.173	0.261	9.689	0.000	0.000	0.135	0.017	0.037	0.103	3.995	0.000	0.000	0.193	0.016	0.035	0.100	3.948	0.000	0.000
Total	WC→YV	−0.024	0.014	−0.040	0.016	−0.823	0.410	0.426	−0.057	0.012	−0.044	0.005	−1.686	0.092	0.090	0.046	0.011	−0.010	0.035	1.035	0.301	0.317
	CR→YV	0.352	0.022	0.190	0.278	10.354	0.000	0.000	0.191	0.018	0.063	0.133	5.343	0.000	0.000	0.215	0.018	0.041	0.110	4.116	0.000	0.000

^1^ *N* = 1988; ^2^ *N* = 1986; ^3^ *N* = 799; YV = youth violence; WC = parental warmth-communication; CR = parental criticism-rejection; MJ = moral justification; EL = euphemistic language; AC = advantageous comparison; DR = displacement of responsibility; DC = distorting consequences; DH = dehumanization; AB = attribution of blame.

## Data Availability

The data of this research are available upon request to the corresponding author.
